# Metformin and Dapagliflozin Attenuate Doxorubicin-Induced Acute Cardiotoxicity in Wistar Rats: An Electrocardiographic, Biochemical, and Histopathological Approach

**DOI:** 10.1007/s12012-023-09784-8

**Published:** 2023-02-15

**Authors:** Shakta Mani Satyam, Laxminarayana Kurady Bairy, Prakashchandra Shetty, P. Sainath, Sanjay Bharati, Akheruz Zaman Ahmed, Varun Kumar Singh, A. J. Ashwal

**Affiliations:** 1 Department of Pharmacology, RAK College of Medical Sciences, RAK Medical and Health Sciences University, Ras Al Khaimah, UAE; 2 Department of Anatomy, Faculty of Medicine, Manipal University College Malaysia, Melaka, Malaysia; 3 Department of Perfusion Technology, Manipal College of Health Professions, Manipal Academy of Higher Education, Manipal, Karnataka India; 4 Department of Nuclear Medicine, Manipal College of Health Professions, Manipal Academy of Higher Education, Manipal, Karnataka India; 5 Department of Anatomy, Melaka Manipal Medical College, Manipal Academy of Higher Education, Manipal, Karnataka India; 6 Department of Pathology, Kasturba Medical College, Manipal Academy of Higher Education, Manipal, Karnataka India; 7 Sahyadri Narayana Multispecialty Hospital, Shimoga, Karnataka India

**Keywords:** Cardiotoxicity, Antidiabetic drugs, Chemotherapy, Chemoprotectant, Electrocardiograph, Cardio-oncology

## Abstract

Doxorubicin is a widely used anticancer drug whose efficacy is limited due to its cardiotoxicity. There is no ideal cardioprotection available against doxorubicin-induced cardiotoxicity. This study aimed to investigate the anticipated cardioprotective potential of metformin and dapagliflozin against doxorubicin-induced acute cardiotoxicity in Wistar rats. At the beginning of the experiment, cardiac screening of experimental animals was done by recording an electrocardiogram (ECG) before allocating them into the groups. Thereafter, a total of thirty healthy adult Wistar rats (150–200 g) were randomly divided into five groups (*n* = 6) and treated for eight days as follows: group I (normal control), group II (doxorubicin control), group III (metformin 250 mg/kg/day), group IV (metformin 180 mg/kg/day), and group V (dapagliflozin 0.9 mg/kg/day). On the 7th day of the treatment phase, doxorubicin 20 mg/kg was administered intraperitoneal to groups II, III, IV, and V. On the 9th day (immediately after 48 h of doxorubicin administration), blood was collected from anesthetized animals for glucose, lipid profile, CK-MB & AST estimation, and ECG was recorded. Later, animals were sacrificed, and the heart was dissected for histopathological examination. We found that compared to normal control rats, CK-MB, AST, and glucose were significantly increased in doxorubicin control rats. There was a significant reversal of doxorubicin-induced hyperglycemia in the rats treated with metformin 250 mg/kg compared to doxorubicin control rats. Both metformin (180 mg/kg and 250 mg/kg) and dapagliflozin (0.9 mg/kg) significantly altered doxorubicin-induced ECG changes and reduced the levels of cardiac injury biomarkers CK-MB and AST compared to doxorubicin control rats. Metformin and dapagliflozin protected the cellular architecture of the myocardium from doxorubicin-induced myocardial injury. Current study revealed that both metformin and dapagliflozin at the FDA-recommended antidiabetic doses mitigated doxorubicin-induced acute cardiotoxicity in Wistar rats. The obtained data have opened the perspective to perform chronic studies and then to clinical studies to precisely consider metformin and dapagliflozin as potential chemoprotection in the combination of chemotherapy with doxorubicin to limit its cardiotoxicity, especially in patients with comorbid conditions like type II diabetes mellitus.

## Introduction

Cancer survival rates are rising exponentially, owing to advances in cancer screening, diagnostic imaging, and therapy methods. However, treatment-related side effects are associated with a higher cancer survival rate, which can significantly influence a patient's health and quality of life [1]. Doxorubicin is an anthracycline anticancer antibiotic used to treat many cancers, including breast cancer and lymphoma [2–4]. It is more likely to produce cardiotoxicity, which is a major cause of morbidity and mortality in cancer patients [5]. Doxorubicin’s therapeutic potential is limited by its cardiotoxicity [6]. Dose-dependent cardiotoxicity caused by doxorubicin can occur at any point throughout treatment and can linger for years after treatment is completed [7]. Because of the various definitions of cardiotoxicity and the wide range of disorders caused by this chemotherapeutic agent, the incidence of doxorubicin-induced cardiotoxicity varies significantly between studies [8–10].

The mechanisms of doxorubicin's therapeutic advantages on tumor cells are distinct from those of its cardiotoxicity. Because of their significant reliance on oxidative substrate metabolism, cardiomyocytes are substantially more vulnerable to the oxidative stress generated by this agent. According to many studies, the primary mechanism for doxorubicin-induced cardiotoxicity is oxidative stress [11–13]. Reactive oxygen species are produced as doxorubicin is oxidized to semiquinone, an unstable metabolite that is then transformed back into doxorubicin [14]. This agent creates an overabundance of reactive oxygen species (ROS) in the mitochondria, resulting in oxidative damage to biological macromolecules such as lipids, proteins, and DNA and affecting the structure and function of cardiac cell membranes [15]. As cardiomyocytes have lower defenses from antioxidant enzymes, doxorubicin decreases endogenous antioxidants and increases lipid peroxidation, altering cardiac function and other toxicities, according to Singal et al. [16]. It has been shown to cause hyperglycemia by increasing insulin resistance. [17–19]. The risk of cardiovascular death in doxorubicin-treated individuals is higher than the chance of tumor recurrence [19]. Doxorubicin has been shown to impair AMPK-alpha (adenosine monophosphate-activated protein kinase-alpha) signaling in the heart, resulting in cellular energy shortages [20]. Doxorubicin can be converted to doxorubicinol, a metabolite that affects the control of iron and calcium by the sarcoplasmic reticulum's calcium pump (ATP2A2) and the sarcolemma's Na^+^/K^+^ pump (RYR2) as well as the mitochondria's F0F1 proton pump [21]. AKR1C3, AKR1A1, CBR1, and CBR3 are potential candidate genes for the synthesis of doxorubicinol [14]. Nitric oxide synthases as well as the NADPH oxidase complex genes NCF4, CYBA, and RAC2 are candidate genes for the production of reactive oxygen species or reactive nitrogen species from the metabolism of doxorubicin [22, 23]. Doxorubicin metabolism within the mitochondria can impair respiration and cause the release of cytochrome-C, which starts apoptosis [24]. Doxorubicin has been reported to interact negatively with phenytoin and cyclosporine. Drug interactions causing cardiotoxicity from co-treatment with doxorubicin and trastuzumab or taxanes such as paclitaxel and docetaxel are more clinically significant [25, 26].

Dexrazoxane is occasionally used in most cancer patients to suppress cardiotoxicity induced by anthracycline.

It may protect against cardiotoxicity by sequestering iron and reducing the production of free radicals [27]. The usage was limited based on concerns that dexrazoxane might increase the risk of second primary malignancies, myelosuppression, and infection, particularly in children. The review of the recent evidence leads to reassessing the European label for dexrazoxane found that it is an effective cardioprotectant in children and adolescents and is not associated with an increased risk of second primary malignancies. It may also be may be associated with specific but reversible toxicities, including myelosuppression [28]. Dexrazoxane side effects frequently include dose-limiting myelotoxicity, which is quite similar to the anthracycline side-effect profile. Therefore, it can be difficult to discern between the side effects of anthracycline treatment and those of dexrazoxane usage. This could also cause testicular atrophy which leads to infertility in males based on its effects on a repeated dose [29]. Due to these serious adverse effects of dexrazoxane, a novel cardioprotective agent is necessary to mitigate doxorubicin-induced cardiotoxicity. An important area of anthracycline research has been figuring out how to keep effectiveness while lowering toxicity.

There is no ideal cardioprotectant readily available against doxorubicin-induced cardiotoxicity and no ideal drug which can be used for both malignancies and diabetes. In this attempt, we did a literature review on the pleiotropic effects of metformin and dapagliflozin. Metformin is a well-established oral antihyperglycemic drug used to treat type II diabetes mellitus. Many in vitro and in vivo studies have reported that metformin exerts a cardioprotective role by activating the AMPK pathway [30–35]. Metformin enhances vascular functioning, which reduces cardiovascular events and death as a result, according to one study [36]. Whittington et al. reported that metformin improves myocardial function by reducing oxidative stress and cardiac fibrosis [37]. Dapagliflozin is a sodium-glucose cotransporter 2 inhibitor that is used to treat type 2 diabetes. Dapagliflozin appears to reduce cardiac fibrosis and improve cardiac function in diabetic rats and reduce glucose reabsorption and promote blood glucose excretion to the urine [38, 39]. Dapagliflozin was found to reduce mortality and the risk of heart failure in type 2 diabetes individuals in the dapagliflozin-Heart Failure (DAPA-HF) trial [40]. The key mechanisms underpinning their cardioprotective effect are improvements in cardiac cell metabolism, ventricular loading circumstances, blockage of Na^+^/H^+^ exchange in myocardial cells, an increase in cytokine generation, and a decrease in cardiac cell necrosis and fibrosis [41].

The cardioprotective potential of metformin and dapagliflozin has been reported at a quite higher dose than the US FDA-recommended maximum dose for the treatment of type II diabetes mellitus. Considering the already defined adverse effects associated with the US FDA-recommended dose of metformin and dapagliflozin, it is not advisable to use these drugs beyond the recommended dose to achieve cardioprotection. At the pre-clinical stage, the cardioprotective potential of these drugs needs to be investigated following the conversion of the recommended human dose to rat dose as per the body surface area ratio given by Paget and Barnes [42]. The combined impact of metformin and dapagliflozin at human equivalent doses against doxorubicin-induced cardiotoxicity has yet to be explored. So, we aimed to investigate the anticipated cardioprotective potential of metformin and dapagliflozin at their normal antidiabetic doses against doxorubicin-induced acute cardiotoxicity in Wistar rats.

## Materials and Methods

### Animals

In this experiment, female Wistar rats weighing 150–200 g were employed. They were kept in separate polypropylene cages under conventional circumstances, which included a temperature of 22–24 °C, a 12-h light/12-h dark cycle, and relative air humidity of 40–60%. The rats received constant access to a regular calorie standard rat pellet diet and tap water (Hindustan Lever Ltd., Mumbai, India). The rats were acclimatized to the laboratory conditions for one week before the experiment began after being randomly assigned to different groups. Fasted animals were deprived of food for 10 h but given unlimited access to water. The Institutional Animal Ethics Committee (IAEC/KMC/33/2021) accepted the experimental protocol, and the studies were carried out by the Government of India's Committee for Control and Supervision of Experiments on Animals (CPCSEA) guidelines.

### Drugs and Reagents

Sigma-Aldrich-Merck Ltd., Bangalore, provided active pharmaceutical ingredient versions of doxorubicin, metformin, and dapagliflozin (India). ASPEN Laboratories Ltd., New Delhi, provided assay kits for lipid profile, creatine kinase-MB (CK-MB), and aminotransferase (AST) quantification (India). This research employed only analytical-grade compounds.

### The Rationale for a Dose Selection of Doxorubicin

Doxorubicin is commonly used to treat a variety of malignancies at a therapeutic dose of 60–75 mg/m^2^ IV once every 21 days. In rats, this dose is similar to 20–25 mg/kg [43].

### The Rationale for a Dose Selection of Metformin

The maximum daily dose of metformin recommended by the US Food and Drug Administration to treat type 2 diabetes patients is 2000 mg per day. According to Paget and Barne's dose conversion table for surface area ratios of certain common laboratory species and man, this dose is equivalent to 180 mg/kg/day in rats [42].

### The Rationale for a Dose Selection of Dapagliflozin

According to the US Food and Drug Administration (US FDA guideline), the dose of dapagliflozin for type 2 diabetes patients is 10 mg per day. According to Paget and Barne's dose conversion table for surface area ratios of certain common laboratory species and man, this dose is equivalent to 0.9 mg/kg/day in rats [42].

### The Rationale for Use of Gum Acacia and Normal Saline (0.9% NaCl)

Gum acacia is a pharmacologically inert substance used widely as emulsifying and tablet binding agent. We dissolved the powder of our test drugs in 2% gum acacia so that while administering these drugs to rats orally, the drug particles remain intact in the feeding needle. Doxorubicin powder is soluble in normal saline (0.9% NaCl). Therefore, we have used gum acacia and normal saline (0.9% NaCl) in control groups to rule out their therapeutic effects.

### Experimental Design

Animals having depressed ST segment/absence of P-wave/inverted P-wave/nonspecific ST-segment/ST-segment elevation were removed from the experiment because of pre-existing cardiac problems and diabetes mellitus. Following that, 30 healthy adult female Wistar rats were randomly divided into five groups and treated for eight days as follows:

#### Group I (Normal Healthy Control)

2% gum acacia; 1 mL/kg/day orally for 8 days + 0.9% NaCl; 1 mL/kg (single dose); i.p. on 7th day.

#### Group II (Doxorubicin Control)

2% gum acacia; 1 mL/kg/day orally for 8 days + doxorubicin; 20 mg/kg (single dose); i.p. on 7th day.

#### Group III (Test-Doxorubicin + Metformin 250 mg/kg)

Metformin; 250 mg/kg/day orally for 8 days + doxorubicin; 20 mg/kg (single dose); i.p. on 7th day.

#### Group IV (Test-Doxorubicin + Metformin 180 mg/kg)

Metformin; 180 mg/kg/day orally for 8 days + doxorubicin; 20 mg/kg (single dose); i.p. on 7th day.

#### Group V (Test-Doxorubicin + Dapagliflozin 0.9 mg/kg)

Dapagliflozin; 0.9 mg/kg/day orally for 8 days + doxorubicin; 20 mg/kg (single dose); i.p. on 7th day.

On the ninth day (immediately after 48 h of doxorubicin administration), all the experimental animals were anesthetized by administering intraperitoneal ketamine (60 mg/kg) and xylazine (10 mg/kg).

### Estimation of Fasting Blood Glucose

Fasting blood samples were taken from the tail vein (tail tip) of rats for blood glucose determination using glucose oxidase–peroxidase reactive strips and a glucometer (Accu-chek, Roche Diagnostics, USA) [44].

### ECG (Electrocardiogram) Recording

In this study, ECG was recorded at two stages. At the beginning of the experiment, cardiac screening of experimental animals was done by recording an ECG before allocating them into the groups. Later, it was recorded on the 9th day (immediately after 48 h of doxorubicin administration). Each rat was anesthetized and placed on an animal surgery table for ECG recording. Electrodes were attached to the palmer surface of rats' shaved limbs. A conductive ECG gel was carefully put over each electrode to avoid the formation of a gel bridge between them. The ECG was recorded for one minute for each animal, and the analysis only used the average of data from 11 consecutive ECG signals. Each experimental animal's ECG was examined qualitatively and quantitatively, and an interventional cardiologist double-checked the results. The PR interval, QT interval, QTc interval, and QRS complex amplitude were all measured in the ECG. The P-wave and ST segment were qualitatively evaluated [43]. The interventional cardiologist validated the results of ECG recording in rats.

### Blood Collection and Serum Preparation

Following ECG recording, capillary tubes were used to collect 2 mL of blood from each sedated rat's retro-orbital venous plexus. Blood was preserved in microcentrifuge tubes, and serum was obtained by centrifuging the entire blood at 3000 rpm for 20 min at 4 °C using a Remi C-24 refrigerated centrifuge following clot formation. The serum was kept at 80 °C for additional biochemical analysis.

### Estimation of Lipid Profile, CK-MB, and AST

Lipid profile, CK-MB, and AST were calculated using a semi-automated analyzer (Star 21 Plus, Mumbai, India) according to the standard technique included with the commercially available kits.

### Heart Isolation, Gross Examination, and Histopathological Analysis

Animals were sacrificed according to the Committee for the Purpose of Control and Supervision on Experiments on Animals (CPCSEA) standards for Laboratory Animal Facility, annexure-6 of euthanasia of laboratory animals. A surgical scalpel was used to make an incision in the thoracic cavity, and the heart was collected from the mediastinum by dissecting it out from the major blood vessels. Following gross examination, it was preserved in 10% formalin for further histopathological evaluation. Following a 24-h fixation in 10% formalin, tissue samples were dehydrated in 50% ethanol for 48 h, 70% ethanol for 24 h, 90% ethanol for 24 h, and 100% ethanol for 24 h. Following that, heart tissues were maintained in xylene until they became translucent. Using embedding rings, tissues were embedded in paraffin wax to make the block, and tissue blocks were stored at 18 °C for 24 h. Then, using a rotary microtome, 5-μm-thick histological sections were taken. The sectioned tissues were stored in a water bath until they were ready to be mounted on lysine-coated slides. After that, the slides with tissues were dried on a hot plate and stained with Haematoxylin and Eosin (H & E). All the slides were observed for the presence of intermuscular edema, cardiomyocyte degeneration, infiltration with inflammatory cells, and myofibrillar loss. From each slide, 10 different fields were observed at ×400 magnification under the light microscope, and histopathological scoring was done as follows—absent (0), mild (1), moderate (2), and severe (3) [45]

### Statistical Analysis

Normally distributed data were reported in terms of mean and standard deviation and analyzed using one-way analysis of variance (ANOVA) followed by post hoc Tukey's test using the Statistical Package for the Social Sciences (SPSS version 16.0). A significance level of *p* ≤ 0.05 was judged statistically significant.

## Results

### Effect on Fasting Blood Glucose Level and Lipid Profile

**Fig. 1:**
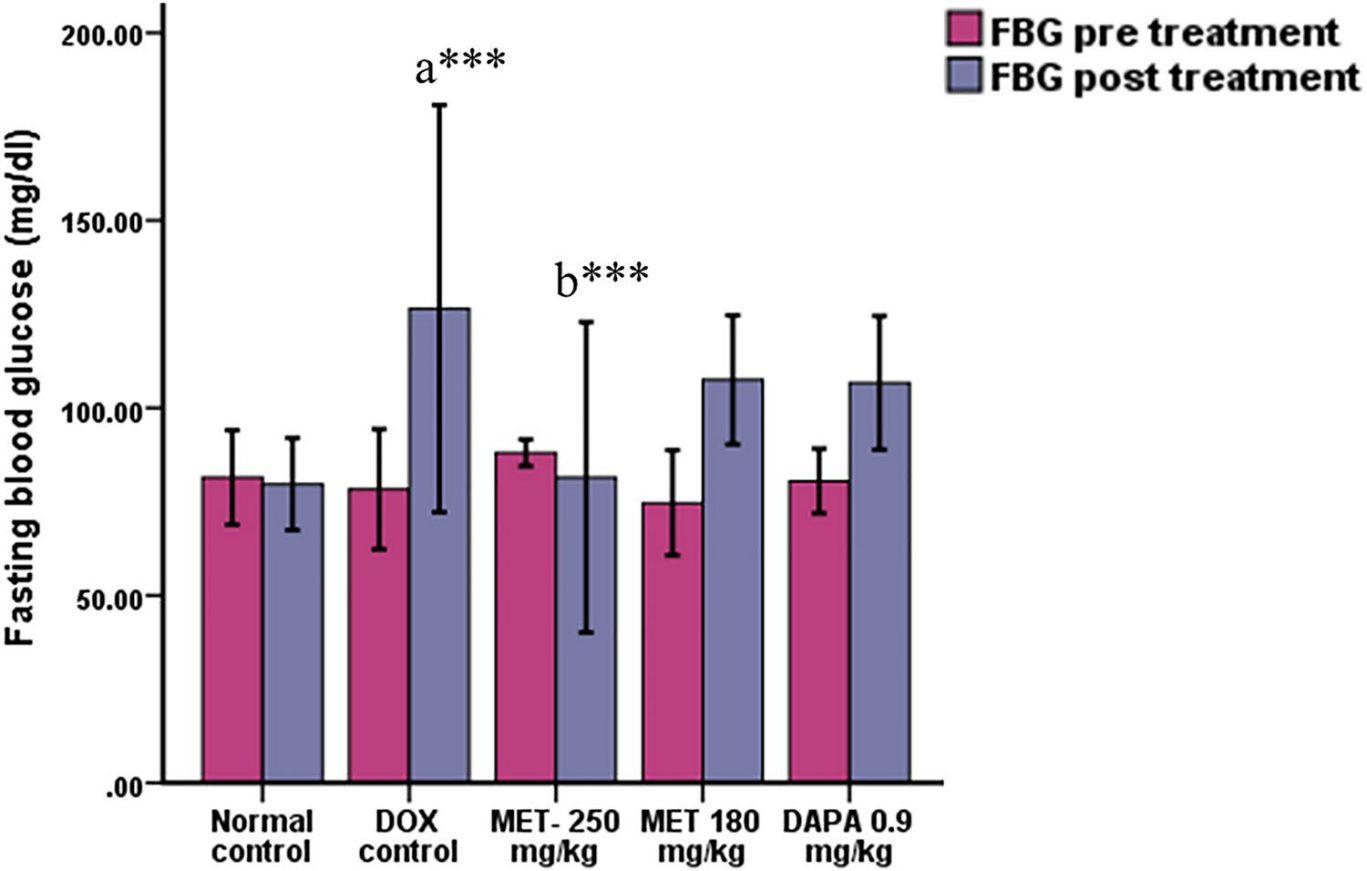
Effect on fasting blood glucose level (Mean ± SD). *a* Compared to normal control, *b* compared to doxorubicin control; *** *p* ≤ 0.001

Fasting blood glucose was significantly increased in the doxorubicin control group (*p* < 0.001) in comparison with the normal control group. Metformin at the dose of 250 mg/kg significantly (*p* = 0.001) prevented the increase in glucose levels compared to the doxorubicin control group (Fig. 1). We did not find any significant changes in the lipid profile of doxorubicin-administered animals compared to the normal control.

### Effect on Cardiac Injury Biomarkers (CK-MB and AST)

**Fig. 2:**
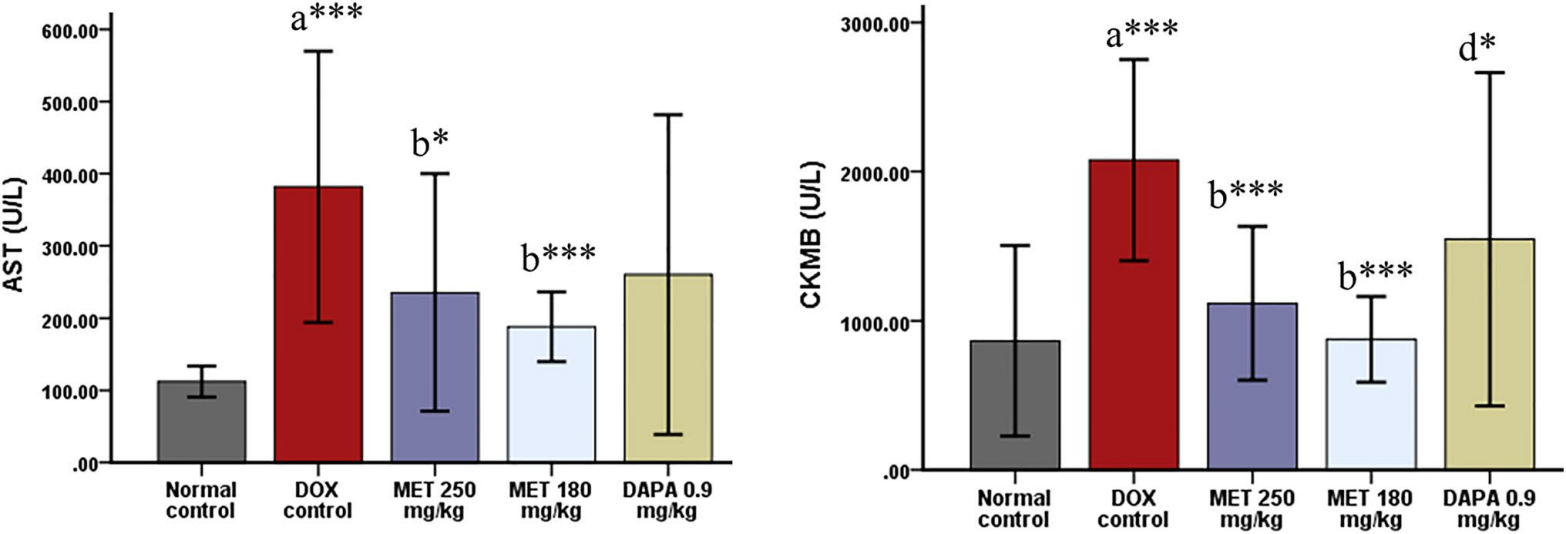
Effect on cardiac injury biomarkers-CK-MB and AST (Mean ± SD). *a* Compared to normal control, *b* compared to doxorubicin control, *d* compared to doxorubicin + metformin 180 mg/kg; ***p ≤ 0.001 and *p ≤ 0.05

There was a significant upsurge in CK-MB (*p* < 0.001) and AST (*p* < 0.001) levels among doxorubicin control rats compared to the normal control group. CK-MB was significantly reduced by both the doses of metformin (*p* < 0.001) and dapagliflozin 0.9 mg/kg (*p* = 0.022) in comparison with the doxorubicin control rats. AST was significantly decreased among metformin 250 mg/kg (*p* = 0.019) and 180 mg/kg (*p* = 0.001) treated rats compared to doxorubicin control rats (Fig. 2).

### Effect on ECG

**Fig. 3:**
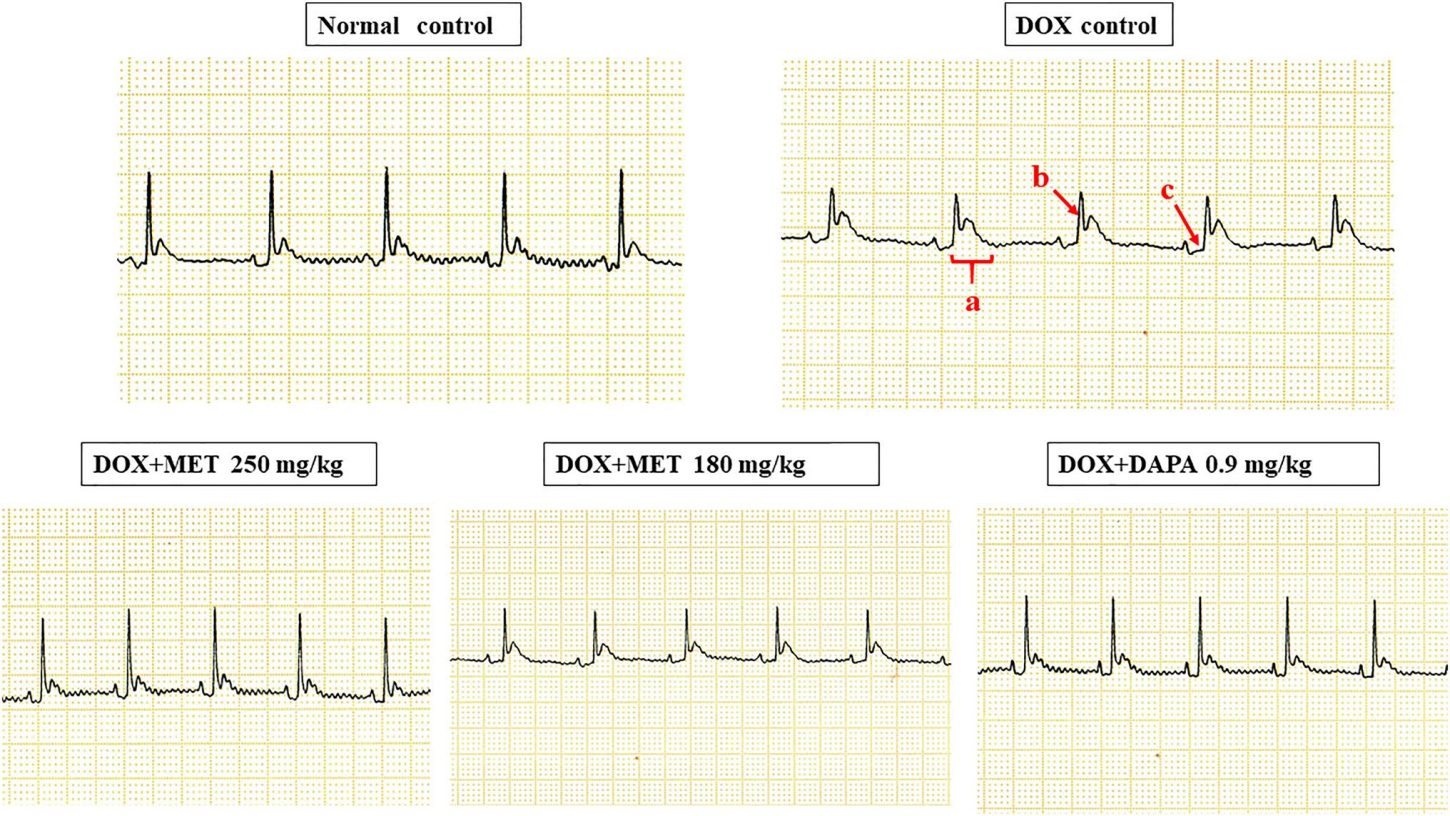
Qualitative analysis of ECG. *a* prolongation of the QT interval, *b* reduced QRS complex amplitude, *c* increased PR interval

**Fig. 4:**
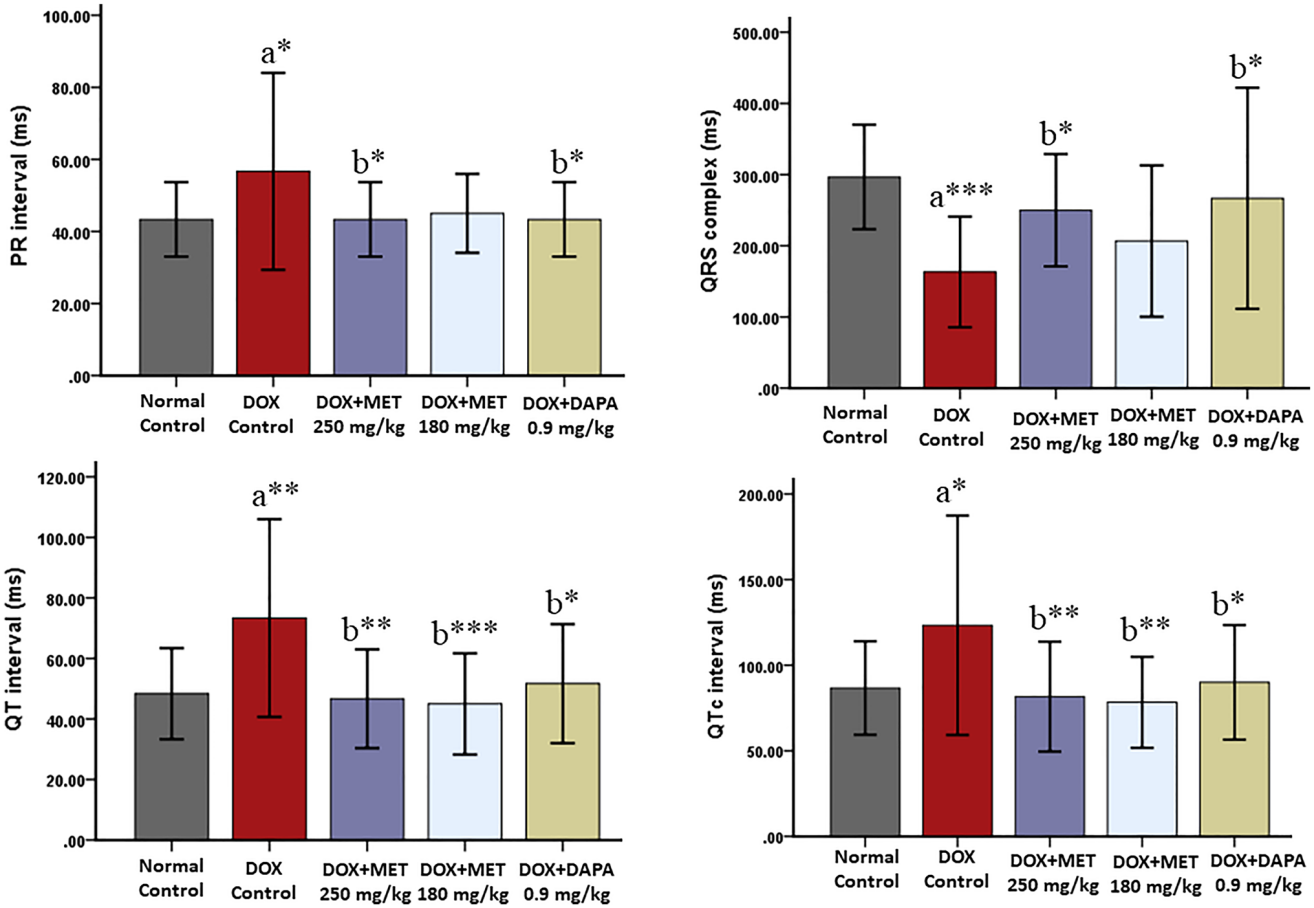
Quantitative analysis of ECG (Mean ± SD). *a* compared to normal control, *b* compared to doxorubicin control; ***p ≤ 0.001, **p ≤ 0.01, and *p ≤ 0.05

The doxorubicin control group exhibited significant prolongation of QT (*p* = 0.003), QTc (*p* = 0.026), and PR (*p* = 0.044) interval and reduction in QRS complex amplitude (*p* = 0.001) compared to normal control rats. QT interval was significantly decreased among the metformin 250 mg/kg (*p* = 0.002), metformin 180 mg/kg (*p* = 0.001), and dapagliflozin 0.9 mg/kg (*p* = 0.012) treated rats in comparison with doxorubicin control rats. Metformin 250 mg/kg (*p* = 0.009), metformin 180 mg/kg (*p* = 0.004), and dapagliflozin 0.9 mg/kg (*p* = 0.050) treated rats exhibited a significant reduction in QTc interval compared to the doxorubicin control group. There was a significant decrease in PR interval for metformin 250 mg/kg (*p* = 0.044) and dapagliflozin 0.9 mg/kg (*p* = 0.044) groups compared to doxorubicin control rats. QRS complex amplitude was significantly improved in metformin 250 mg/kg (*p* = 0.050) and dapagliflozin 0.9 mg/kg (*p* = 0.015) groups compared to the doxorubicin control group (Figs. 3, 4).

### Gross Examination of Isolated Hearts

Ischemic changes were observed in the hearts of doxorubicin control rats as pale/yellow with hyperemic or hemorrhagic borders/white–gray (scar), but these appearances were missing in normal control and the metformin/dapagliflozin-treated groups.

### Histopathological Examination of the Heart

**Fig. 5:**
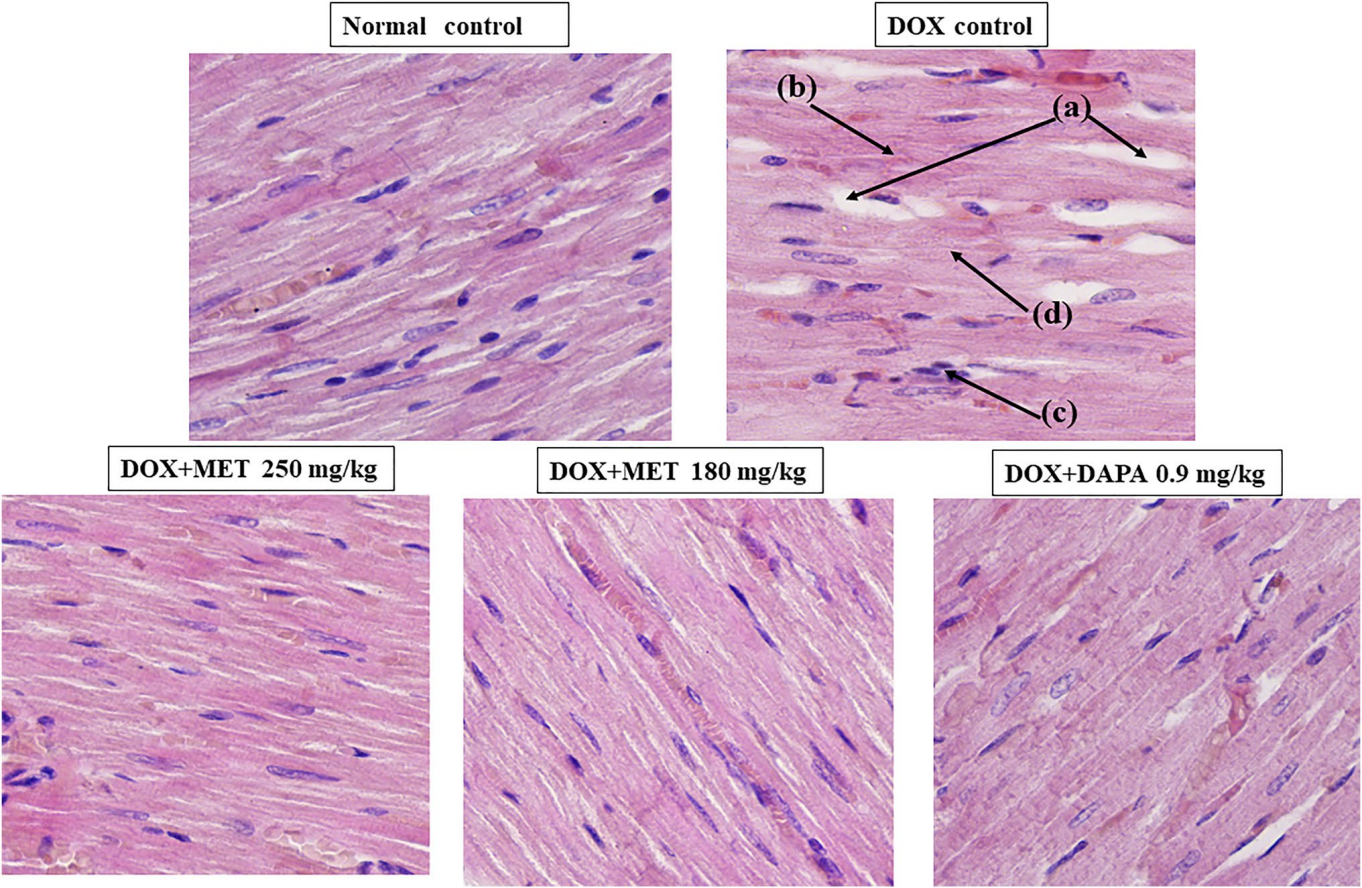
Histopathological examination of the heart (longitudinal Sect. 400X). *a* intermuscular edema, b- cardiomyocytes degeneration, c- infiltration with inflammatory cells, d- myofibrillar loss

Cardiomyocytes from doxorubicin control rats showed significant cardiomyocyte degeneration, intermuscular edema, and mild inflammatory cell infiltration, myofibrillar loss under light microscopy (400 X). These pathological alterations were reduced in metformin and dapagliflozin-treated cardiomyocytes, and their architecture was nearly identical to that of the normal control group (Fig. 5).

### Effect of Metformin and Dapagliflozin on Cardiomyocyte Architecture Seen Under the Light Microscope Following H & E Staining

There was a significant increase in intermuscular edema (p < 0.001), and cardiomyocyte generation (p < 0.001) among the DOX-intoxicated control rats compared to normal healthy control rats (Table 1). These pathological alterations were reduced in metformin and dapagliflozin-treated cardiomyocytes, and their architecture was nearly identical to that of the normal control group (Fig. 5).

**Table 1:** Quantitative scoring of histopathological examination of heart tissues using H & E staining

Groups	Scoring for intermuscular edema (Mean ± SD)	Scoring for cardiomyocytes degeneration (Mean ± SD)
Normal control	0.00 ± 0.00	0.00 ± 0.00
Doxorubicin control	2.33 ± 0.51^***a^	2.16 ± 0.40^***a^
Doxorubicin + MET 250 mg/kg	1.50 ± 0.54^*b^	1.16 ± 0.40^***b^
Doxorubicin + MET 180 mg/kg	1.33 ± 0.51^**b^	1.33 ± 0.51^**b^
Doxorubicin + DAPA 0.9 mg/kg	1.16 ± 0.40^***b^	1.16 ± 0.40^***b^

## Discussion

The present study has demonstrated acute cardiotoxicity with a single dose of doxorubicin administration to the Wistar rats. Doxorubicin binds to the negatively charged phospholipid cardiolipin, which is prevalent in the inner mitochondrial membrane and accumulates in cardiomyocytes’ mitochondria [43]. Doxorubicin causes an overproduction of reactive oxygen species (ROS) in the mitochondria, causing oxidative damage to biological macromolecules such as lipids, proteins, and DNA and disrupting cardiac cell membrane structure and function [46]. Doxorubicin causes acute cardiotoxicity that manifests during and within 2–3 days after a single-dose administration [46, 47].

We observed that doxorubicin has significantly altered ECG waves in the form of an inverted p-wave/increased PR interval/prolonged QT and QTc interval/reduced QRS complex amplitude/nonspecific ST segment. The majority of the ECG changes could be related to a change in membrane function caused by doxorubicin-induced lipid peroxidation. The absence or changed form of the P-wave occurs in a variety of cardiac arrhythmias, the most prevalent of which is atrial fibrillation. [48–50]. The distribution of depolarization from the atria to the ventricles is reflected by the PR interval [51, 52]. To diagnose atrioventricular blockages, the length of the PR interval must be measured. Doxorubicin has been shown to increase the PR interval [53, 54]. Supraventricular arrhythmias are linked to complex QRS narrowing. In cardiac insufficiency, myocardial ischemia, and right and left bundle branch blockages, the big QRS complex shows ventricular rhythms as well as abnormalities in intraventricular conduction. In doxorubicin-treated rats, wide QRS complexes were seen [55]. Due to cancelations and decreased electromotive force generation, several myocardial infarctions have been associated with a reduction in the amplitude of QRS complexes [56–58]. The ST segment is the time between the end of the QRS complex and the start of the T wave, and it reflects the moment when the ventricles depolarize. After myocardial infarction and myocardial ischemia, considerable alterations in the ST segment were identified, but specific criteria for major ST-segment abnormalities were unclear. In reaction to doxorubicin, nonspecific ST alterations have been described [59, 60]. Detecting the ST segment in rat ECG is difficult due to the T wave's continual increase in continuity with the S wave [61]. This parameter's pathological duration reflects heart electrical activity abnormalities produced by intrinsic heart disease or exogenous chemical harmful effects. An extended QT interval [62, 63] is thought to be a good predictor of drug-induced cardiotoxicity. Doxorubicin has been shown to cause QT prolongation in numerous investigations [53, 54, 63, 64]. The length of the QT interval is known to be dependent on heart rate (HR). As the ratio of systole and diastole duration increases, a rise in the HR usually shortens QT. As a result, the corrected QT interval (QTc), which accounts for variations in HR, is frequently utilized as a more objective ventricular depolarization and repolarization metric [66, 67]. Metformin and dapagliflozin were found to significantly counteract DOX-induced PR, QT, and QTc prolongation, as well as a reduction in QRS complex amplitude, in the current investigation. ECG alterations in patients using metformin and dapagliflozin have been reported to be similar. [55, 68–72]. This could be due to their potential to reduce doxorubicin-induced lipid peroxidation and so stabilize membranes.

Myocardial damage is indicated by elevated blood levels of CK-MB and AST [10]. Doxorubicin increased the levels of myocardial damage indicators such as CK-MB and AST in our findings. This is consistent with earlier research that suggests DOX-induced oxidative stress can result in cardiac lipid peroxidation and the release of these enzymes into the bloodstream [20, 73, 74]. Cardiomyocytes from doxorubicin control rats exhibited significant cardiomyocyte degeneration, intermuscular edema but mild inflammatory cell infiltration, and myofibrillar loss. Out of the four prominent histopathological features perceived following myocardial infarction, two features i.e., intermuscular edema and cardiomyocyte degeneration are usually seen during the first 48 h of myocardial infarction, and the other two features i.e., inflammatory cell infiltration and myofibrillar loss are seen gradually in a time-dependent fashion later on. Myocardial infarction is evident in the current study from ECG and biochemical perspectives. It has been commonly observed that structural changes are seen following biochemical alterations.

Both metformin and dapagliflozin significantly reduced the levels of cardiac damage indicators CK-MB, AST, and histological changes in doxorubicin-treated rats, according to our findings. Doxorubicin-induced cardiotoxicity has been linked to a variety of pathways [55, 75]. Its inhibitory effects on AMPK, on the other hand, have been thoroughly described [55, 76].

Metformin has been shown to activate AMPK, resulting in increased glucose absorption and stimulation of glycolysis in cardiomyocytes. AMPK activation also inhibits cell growth and proliferation, which aids in the prevention of cardiac remodeling after a heart attack [77]. Metformin has also been shown to inhibit reactive oxygen species production and oxidative stress caused by doxorubicin [51, 55]. SGLT-2 inhibitors have been found to reduce myocardial oxidative stress, fibrosis, and vascular remodeling which play important roles in the pathogenesis of cardiovascular diseases [71].

In diabetic cardiomyopathy models in mice and rats, heart failure models in zebrafish embryos, and a myocardial ischemia model in rats, SGLT-2 inhibitors have been found to ameliorate cardiac histopathologic alterations [78]. Dapagliflozin has been shown to reduce the augmentation of mitochondrial reactive oxygen species generation, depolarization, and edema in a cardiac ischemia–reperfusion injury model [79–81]. PGC1- and CPT1 are mitochondrial metabolism-related proteins in the heart that play important roles in fatty acid oxidation [30]. Dapagliflozin therapy enhanced CPT1 protein expression and boosted complex I of the ETC expression, indicating that it prevents myocardial energy metabolism depletion when I/R damage occurs [71]. When combined with existing guideline-directed medical therapy, sodium-glucose cotransporter 2 (SGLT2) inhibitor dapagliflozin has been shown to minimize hospitalization for heart failure or mortality linked with cardiovascular causes in recent randomized controlled trials, including DAPA-HF [82–84]. According to one study, dapagliflozin treatment for patients with ST-segment elevation myocardial infarction (STEMI) and type 2 diabetes mellitus after percutaneous coronary intervention (PCI) can improve cardiac function, reduce inflammation, and lower the risk of adverse cardiovascular outcomes [85]. Metformin and dapagliflozin both decreased doxorubicin-induced myocardial damage in our investigation, possibly through the mechanisms outlined above.

## Conclusion

Our findings revealed that both metformin and dapagliflozin at the FDA-recommended antidiabetic doses exert cardioprotection against doxorubicin-induced acute cardiotoxicity in Wistar rats. This research can be elaborated further to investigate potential molecular mechanisms underlying the anticipated optimal cardioprotective action of metformin and dapagliflozin against doxorubicin-induced acute and chronic cardiotoxicity at single and repeated doses.
